# Discriminating Interpatient Variabilities of *RAS* Gene Variants for Precision Detection of Thyroid Cancer

**DOI:** 10.1001/jamanetworkopen.2024.11919

**Published:** 2024-05-17

**Authors:** Guodong Fu, Ronald S. Chazen, Christina MacMillan, Ian J. Witterick

**Affiliations:** 1 Alex and Simona Shnaider Research Laboratory in Molecular Oncology, Lunenfeld-Tanenbaum Research Institute, Mount Sinai Hospital, Sinai Health, Toronto, Ontario, Canada; 2 Department of Pathology and Laboratory Medicine, Mount Sinai Hospital, Sinai Health and University of Toronto, Toronto, Ontario, Canada; 3 Joseph and Mildred Sonshine Family Centre for Head and Neck Diseases, Mount Sinai Hospital, Sinai Health, Toronto, Ontario, Canada; 4 Department of Otolaryngology-Head and Neck Surgery, Mount Sinai Hospital, Sinai Health and University of Toronto, Toronto, Ontario, Canada

## Abstract

**Importance:**

Interpatient variabilities in genomic variants may reflect differences in tumor statuses among individuals.

**Objectives:**

To delineate interpatient variabilities in *RAS* variants in thyroid tumors based on the fifth World Health Organization classification of thyroid neoplasms and assess their diagnostic significance in cancer detection among patients with thyroid nodules.

**Design, Setting, and Participants:**

This prospective diagnostic study analyzed surgically resected thyroid tumors obtained from February 2016 to April 2022 and residual thyroid fine-needle aspiration (FNA) biopsies obtained from January 2020 to March 2021, at Mount Sinai Hospital, Toronto, Ontario, Canada. Data were analyzed from June 20, 2022, to October 15, 2023.

**Exposures:**

Quantitative detection of interpatient disparities of *RAS* variants (ie, *NRAS, HRAS*, and *KRAS*) was performed along with assessment of *BRAF* V600E and *TERT* promoter variants (C228T and C250T) by detecting their variant allele fractions (VAFs) using digital polymerase chain reaction assays.

**Main Outcomes and Measures:**

Interpatient differences in *RAS*, *BRAF* V600E, and *TERT* promoter variants were analyzed and compared with surgical histopathologic diagnoses. Malignancy rates, sensitivity, specificity, positive predictive values, and negative predictive values were calculated.

**Results:**

A total of 438 surgically resected thyroid tumor tissues and 249 thyroid nodule FNA biopsies were obtained from 620 patients (470 [75.8%] female; mean [SD] age, 50.7 [15.9] years). Median (IQR) follow-up for patients who underwent FNA biopsy analysis and subsequent resection was 88 (50-156) days. Of 438 tumors, 89 (20.3%) were identified with the presence of *RAS* variants, including 51 (11.6%) with *NRAS*, 29 (6.6%) with *HRAS*, and 9 (2.1%) with *KRAS*. The interpatient differences in these variants were discriminated at VAF levels ranging from 0.15% to 51.53%. The mean (SD) VAF of *RAS* variants exhibited no significant differences among benign nodules (39.2% [11.2%]), noninvasive follicular thyroid neoplasm with papillary-like nuclear features (NIFTPs) (25.4% [14.3%]), and malignant neoplasms (33.4% [13.8%]) (*P* = .28), although their distribution was found in 41.7% of NIFTPs and 50.7% of invasive encapsulated follicular variant papillary thyroid carcinomas (*P* < .001). *RAS* variants alone, regardless of a low or high VAF, were significantly associated with neoplasms at low risk of tumor recurrence (60.7% of RAS variants vs 26.9% of samples negative for RAS variants; *P* < .001). Compared with the sensitivity of 54.2% (95% CI, 48.8%-59.4%) and specificity of 100% (95% CI, 94.8%-100%) for *BRAF* V600E and *TERT* promoter variant assays, the inclusion of *RAS* variants into *BRAF* and *TERT* promoter variant assays improved sensitivity to 70.5% (95% CI, 65.4%-75.2%), albeit with a reduction in specificity to 88.8% (95% CI, 79.8%-94.1%) in distinguishing malignant neoplasms from benign and NIFTP tumors. Furthermore, interpatient differences in 5 gene variants (*NRAS, HRAS, KRAS, BRAF,* and *TERT*) were discriminated in 54 of 126 indeterminate FNAs (42.9%) and 18 of 76 nondiagnostic FNAs (23.7%), and all tumors with follow-up surgical pathology confirmed malignancy.

**Conclusions and Relevance:**

This diagnostic study delineated interpatient differences in *RAS* variants present in thyroid tumors with a variety of histopathological diagnoses. Discrimination of interpatient variabilities in *RAS* in combination with *BRAF* V600E and *TERT* promoter variants could facilitate cytology examinations in preoperative precision malignancy diagnosis among patients with thyroid nodules.

## Key Points

**Question:**

Is discrimination of interpatient variabilities of *RAS* gene variants associated with improved accuracy in malignancy diagnosis among thyroid nodules?

**Findings:**

This diagnostic study of 620 patients, including 438 surgically resected thyroid tumor tissues and 249 thyroid nodule fine-needle aspiration biopsies, delineated interpatient differences in *RAS* variants at the variant allele fraction (VAF) levels, ranging from 0.15% to 51.53%. While *RAS* variants alone, regardless of the extent of variation, were associated with low-risk thyroid cancer in 88.8% of tumor samples, they did not definitively distinguish malignancy of an unknown tumor; however, detection of interpatient variabilities of *RAS*, *BRAF,* and *TERT* promoter variants in combination could assist in classifying indeterminate thyroid nodules.

**Meaning:**

These findings suggest that discrimination of interpatient differences in genomic variants could facilitate precision cancer detection, including preoperative malignancy diagnosis and stratification of low-risk tumors from high-risk ones, among patients with indeterminate thyroid nodules.

## Teaser

This diagnostic study assesses interpatient differences in *RAS* variants and their utility in malignancy detection among patients with thyroid nodules.

## Introduction

Thyroid cancer, especially papillary thyroid cancer (PTC), has experienced a rapid increase in incidence since the 1980s^1^ and is primarily diagnosed through ultrasonographic examinations and fine-needle aspiration (FNA) biopsy of suspicious nodules.^2,3^ However, approximately 30% of FNAs exhibit an indeterminate diagnosis, and 10% of findings are nondiagnostic.^4^ Patients with indeterminate thyroid nodule findings usually undergo diagnostic surgery, with 20% to 30% of nodules being detected as malignant. Thus, up to 70% to 80% of patients with indeterminate nodules found histologically benign have undergone unnecessary surgical procedures. Patients with nondiagnostic cytological findings are typically recommended for a repeat FNA, with 13% of nodules detected as being malignant.^4^ Cancer arises along with genetic alterations. Molecular assays of FNA specimens are being increasingly used to enhance preoperative diagnostic accuracy for patients with indeterminate cytological findings and avoid unnecessary surgery for benign thyroid nodules.^2,5^

*RAS* is the most frequently variated gene family in human cancer. Approximately 19% of patients with cancer harbor activating variations from 3 *RAS* gene isoforms: *NRAS* (OMIM 164790) in 17% of patients, *HRAS* (OMIM 190020) in 7% of patients, or *KRAS* (OMIM 190070) in 75% of patients.^6^ Similarly, *RAS* variants are the second most common alterations in thyroid nodules, with *NRAS* variants being the dominant isoform followed by *HRAS* and *KRAS*.^7,8,9,10^ In thyroid tumors, *RAS* gene variations are detected in tumors spanning a wide spectrum of histological diagnoses, with a prevalence of 10% to 30% in PTC,^11,12,13^ 40% to 50% in follicular thyroid carcinomas (FTCs),^14,15^ 12% to 85% in follicular adenoma or hyperplasia, and 5% to 46% in noninvasive follicular thyroid neoplasm with papillary-like nuclear features (NIFTPs).^14,16^ Indeterminate thyroid nodules carrying *RAS* variants have shown malignancy rates varying from 9% to 83%,^7,8,9,10,17^ and such discrepancies can be primarily attributed to the use of small patient cohorts in these studies. Despite the widespread application of *RAS* variants in panel tests, assays of *RAS* variants often yield inconclusive results in detecting malignancy of thyroid nodules, frequently leading to a diagnostic surgery.^2,12,18^ On the contrary, *BRAF* V600E and *TERT* promoter variants (C228T and C250T) are the most frequently detected genetic variants in thyroid nodules, providing a more definitive basis for cancer diagnosis.^19,20,21^

Interpatient variabilities in genomic variants may reflect differences in tumor statuses among individuals.^20^ However, the diagnostic impact of discriminating interpatient variabilities of *RAS* variants on cancer detection remains unclear, particularly under the 2022 updated fifth World Health Organization (WHO) classification of thyroid neoplasms.^22^ In alignment with the WHO classification, the 2023 Bethesda System for Reporting Thyroid Cytopathology (BSRTC)^23^ has updated nomenclature for each of the 6 diagnostic categories: I, nondiagnostic (ND); II, benign; III, atypia undetermined significance (AUS); IV, follicular neoplasm (FN); V, suspicious for malignancy (SFM); and VI, malignant.^23^ Currently, the methods of detecting *RAS* variations are mainly based on polymerase chain reaction (PCR) and Sanger sequencing or next-generation sequencing (NGS). This study aimed to delineate interpatient disparities of *RAS* variants in thyroid tissues by quantifying variant allele fraction (VAF) using digital PCR (dPCR) assays and to examine their diagnostic associations with the preoperative detection of malignancy among patients with thyroid nodules.

## Methods

This prospective diagnostic study was reviewed and approved by the Sinai Health Research Ethics Bord. All patients provided written informed consent, and samples were deidentified for data analysis. Data are reported in alignment with the Standards for Reporting of Diagnostic Accuracy (STARD) reporting guideline.

### Patients and Clinical Samples

A total of 438 thyroid tissue specimens were obtained from surgically resected thyroid tumors with a maximum dimension of 1 cm or larger from 436 consecutive patients who underwent surgery between February 1, 2016, and April 4, 2022, and 249 FNA specimens were collected from 234 consecutive patients who underwent biopsy procedures between January 22, 2020, and March 2, 2021, at Mount Sinai Hospital, Sinai Health, Toronto, Canada. All surgical tissue specimens sampled were quickly placed in liquid nitrogen and transferred to −80 °C for long-term preservation. As for preoperative biopsies, all FNAs were routinely obtained under ultrasonographic guidance using a 23-gauge needle and subjected to CytoLyte (Hologic) fixation. After cytological examination according to the BSRTC,^4,23^ the leftover materials of a total of 249 FNA biopsies were collected and stored at 4 °C until DNA purification. These preoperative biopsies primarily included ND and indeterminate (BSRTC categories I, III, IV, and V) specimens, along with some malignant (BSRTC category VI) and benign (BSRTC category II) specimens. A follow-up of thyroid nodules was conducted among patients who had previously undergone FNA procedures and subsequently underwent surgery. The patient clinical records, surgical pathology reports, and hematoxylin and eosin–stained sections were reviewed. The final histological diagnoses were made in accordance with the fifth WHO classification of thyroid neoplasms^22^ and Protocol for the Examination of Specimens From Patients With Carcinomas of the Thyroid Gland.^24^ Patients with cancer were further stratified as having low, intermediate, or high risk of recurrence based on the 2015 American Thyroid Association guidelines.^25^

### Droplet dPCR Assays of *RAS*, *BRAF* V600E, and *TERT* promoter variants

Molecular assays for the most prevalent *RAS* variants of 3 *RAS* genes, *NRAS* (Q61R or Q61K), *HRAS* (Q61R or Q61K), and *KRAS* (G12C, G12D, G12V, G12A, or G13D), were developed using locked nucleic acid probe–based droplet dPCR by following the strategy and procedures recently established for the VAF assays of *BRAF* V600E and *TERT* promoter variants (C228T and C250T).^20,26^ The details of DNA extraction, dPCR assays, and verification of *RAS* variants using PCR and Sanger sequencing were documented in the eMethods in Supplement 1.

### Statistical Analysis

Data were summarized as frequencies and percentages for categorical variables and means and SDs for continuous variables. Blinded central review–based 2022 WHO histologic classification and 2023 BSRTC were used as the reference standard.^22,23^ The continuous parametric variables were compared by *t* test or 1-way analysis of variance test. Associations between molecular status and the clinicopathological characteristics were assessed by χ^2^ or Fisher exact test with 95% CIs. Statistical tests were conducted using SPSS software version 22.0 (IBM). *P* values were 2-sided, with *P* < .05 considered statically significant. Data were analyzed from June 20, 2022, to October 15, 2023.

## Results

### Baseline Characteristics of Patients and Thyroid Specimens

**Figure 1.**
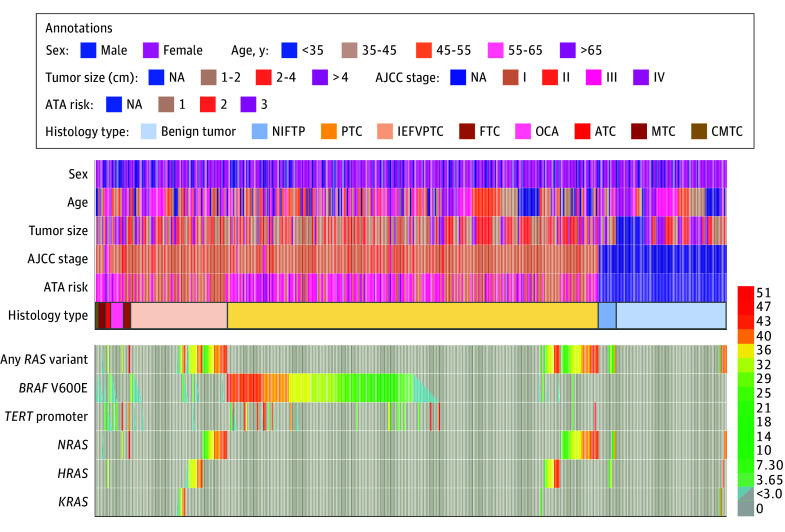
Clinicomolecular Chacteristics of Interpatient Variabilities of *RAS*, *BRAF* V600E, and *TERT* Promoter Variants at the Variant Allele Fraction (VAF) Level in Thyroid Tumors AJCC indicates American Joint Committee on Cancer Cancer Staging Manual, 8th Edition; ATA, American Thyroid Association; ATC, anaplastic thyroid carcinoma; CMTC, cribriform morular thyroid carcinoma; FTC, follicular thyroid carcinomas; IEFVPTC, invasive encapsulated follicular variant papillary thyroid carcinoma; MTC, medullary thyroid carcinomas; NA, not applicable; NIFTP, noninvasive follicular thyroid neoplasm with papillary-like nuclear features; OCA, oncocytic carcinomas of the thyroid; and PTC, papillary thyroid carcinomas.

**Figure 2.**
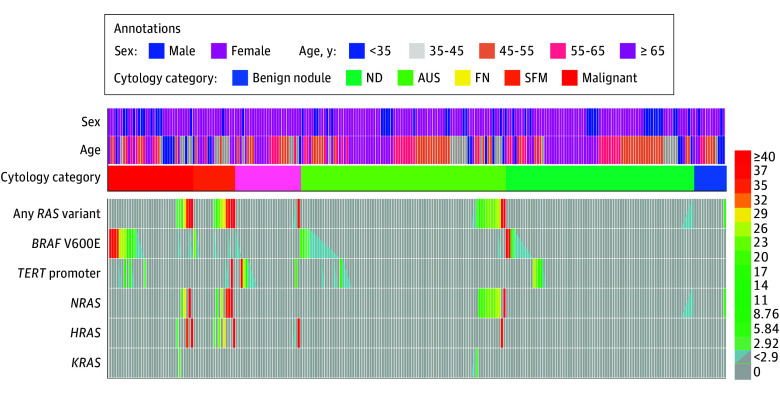
Interpatient Variabilities of *RAS*, *BRAF* V600E, and *TERT* Promoter Variants at the Variant Allele Fraction Level in Residual Fine-Needle Aspiration Specimens AUS indicates atypia of undetermined significance; FN, follicular neoplasm; ND, nondiagnostic; and SFM, suspicious for malignancy.

A total of 438 surgically resected thyroid tumor tissues and 249 thyroid nodule FNA biopsies were obtained from 620 patients (470 [75.8%] female; mean [SD] age, 50.7 [15.9] years). Of 438 thyroid tumors, 431 were follicular cell-derived neoplasms, comprising 77 benign tumors (thyroid follicular nodular disease, follicular adenoma, or oncocytic adenoma), 12 NIFTPs, and 343 malignant neoplasms, including 258 PTCs, 67 invasive encapsulated follicular variant PTCs (IEFVPTCs), 5 FTCs, 10 oncocytic carcinomas of the thyroid (OCAs), and 3 anaplastic thyroid carcinomas (ATCs). The cohort also included 5 medullary thyroid carcinomas (MTCs) and 2 cribriform morular thyroid carcinomas (CMTCs) (Figure 1 and Table 1; eTable 1 in Supplement 1). Hence the surgical tumor cohort exhibited a high tumor malignancy rate of 79.7% (95% CI, 75.5%-83.3%). In a separate cohort of 249 FNA biopsies, there were 76 (30.5%) with ND findings, 126 (50.6%) with indeterminate findings, 34 (13.7%) with malignant findings, and 13 (5.2%) with benign findings (Figure 2 and Table 2; eTable 2 in Supplement 1). The indeterminate FNAs comprised 83 AUS (65.9%), 26 FN (20.6%), and 17 SFM (13.5%).

**Table 1.** Association of VAF Assay Findings of *RAS* Variants With Clinicohistopathologic Features of Thyroid Tumors

Characteristic	Patients, No. (%)			*P* value^a^^,^^b^	Patients with *RAS* VAF>0		*P* value^b^	Patients by *RAS* variant, No. (%)			*P* value^b^
	Total	*RAS* VAF = 0	*RAS* VAF > 0		VAF < 1	VAF ≥ 1		*HRAS*	*KRAS*	*NRAS*	
Sample	438 (100)	349 (79.7)	89 (20.3)	NA	5 (1.1)	84 (19.2)	NA	29 (6.6)	9 (2.1)	51 (11.6)	NA
Sex											
Male	113 (25.8)	93 (26.6)	20 (22.5)	.50	1 (20.0)	19 (22.6)	.80	11 (37.9)	1 (11.1)	8 (15.7)	.11
Female	325 (74.2)	256 (73.4)	69 (77.5)		4 (80.0)	65 (77.4)		18 (62.1)	8 (88.9)	43 (84.3)	
Age, y											
Mean (SD)	49.7 (15.2)	50.3 (15.3)	47.2 (14.4)	.08	39.5 (10.2)	47.7 (14.6)	.11	49.3 (11.7)	50.2 (14.5)	45.5 (15.8)	.21
<55	271 (61.9)	211 (60.5)	60 (67.5)	.27	5 (100)	55 (65.5)	.17	18 (62.1)	7 (77.8)	35 (68.6)	.55
≥55	167 (38.1)	138 (39.5)	29 (32.6)		0	29 (34.5)		11 (37.9)	2 (22.2)	16 (31.4)	
Thyroidectomy											
Partial	178 (40.6)	131 (37.5)	47 (52.8)	.01	2 (40.0)	45 (53.6)	.02	19 (65.5)	3 (33.3)	25 (49.0)	.01
Total	260 (59.4)	218 (62.5)	42 (47.2)		3 (60.0)	39 (46.4)		10 (34.5)	6 (66.7)	26 (51.0)	
Tumor size, cm^c^											
Mean (SD)	3.0 (1.9)	3.0 (1.9)	3.0 (1.9)	.98	3.1 (1.7)	3.0 (1.9)	.98	2.8 (2.3)	2.4 (0.9)	3.2 (1.7)	.58
1-2	170 (40.7)	137 (41.6)	33 (37.1)	.13	2 (40.0)	31 (36.9)	.35	13 (44.8)	4 (44.4)	16 (31.4)	.31
2-4	156 (37.3)	115 (35.0)	41 (46.1)		2 (40.0)	39 (46.4)		12 (41.4)	4 (55.6)	24 (47.1)	
>4	92 (22.0)	77 (23.4)	15 (16.9)		1 (20.0)	14 (16.7)		4 (13.8)	0	11 (21.6)	
ETE^d^											
None	310 (88.8)	232 (85.9)	78 (98.7)	.002	5 (100)	73 (98.6)	.002	25 (96.2)	7 (100)	46 (100)	.008
Identified	39 (11.2)	38 (14.1)	1 (1.3)		0	1 (1.4)		1 (3.8)	0	0	
LNM^d^											
None	240 (68.8)	165 (61.1)	75 (94.9)	<.001	4 (80.0)	71 (95.9)	<.001	25 (96.2)	6 (85.7)	44 (95.7)	<.001
Identified	109 (31.2)	105 (38.9)	4 (5.1)		1 (0.9)	3 (4.1)		1 (3.8)	1 (14.3)	2 (4.3)	
Capsular invasion^d^											
None	254 (72.8)	221 (81.9)	33 (41.8)	<.001	3 (60.0)	30 (40.5)	<.001	11 (42.3)	3 (42.9)	19 (41.3)	<.001
Identified	95 (27.2)	49 (18.1)	46 (58.2)		2 (40.0)	44 (59.8)		15 (57.7)	4 (57.1)	27 (58.7)	
Lymphatic invasion^d^											
None	243 (69.6)	169 (62.6)	74 (93.7)	<.001	3 (60.0)	71 (95.9)	<.001	26 (100)	6 (85.7)	42 (91.3)	<.001
Identified	106 (30.4)	101 (37.4)	5 (6.3)		2 (40.0)	3 (4.1)		0	1 (14.3)	4 (8.7)	
Perineural invasion^d^											
None	322 (92.3)	244 (90.4)	78 (98.7)	.01	5 (100)	73 (98.6)	.03	25 (96.2)	7 (100)	46 (100)	.08
Identified	27 (7.7)	26 (9.6)	1 (1.3)		0	1 (1.4)		1 (3.8)	0	0	
ATA malignant risk^d^											
0	89 (20.4)	79 (22.6)	10 (11.2)	<.001	0	10 (11.9)	<.001	3 (10.3)	2 (22.2)	5 (9.8)	<.001
1	148 (33.8)	94 (26.9)	54 (60.7)		2 (40.0)	52 (61.9)		21 (72.4)	6 (66.7)	27 (52.9)	
2	201 (45.9)	176 (50.4)	25 (28.1)		3 (60.0)	22 (26.2)		5 (17.2)	1 (11.1)	19 (37.3)	
Histology											
Benign	77 (17.6)	72 (20.6)	5 (5.6)	<.001	0	5 (6.0)	<.001	1 (3.4)	2 (22.2)	2 (3.9)	<.001
NIFTP	12 (2.7)	7 (2.0)	5 (5.6)		0	5 (6.0)		2 (6.9)	0	3 (5.9)	
PTC	258 (58.9)	217 (62.2)	41 (46.1)		3 (60.0)	38 (45.2)		12 (41.4)	2 (22.2)	27 (52.9)	
IEFVPTC	67 (15.3)	33 (9.5)	34 (38.2)		2 (40.0)	32 (38.1)		12 (41.4)	5 (55.6)	17 (33.3)	
FTC	5 (1.1)	4 (1.1)	1 (1.1)		0	1 (1.2)		0	0	1 (2.0)	
OCA	10 (2.3)	9 (2.6)	1 (1.1)		0	1 (1.2)		0	0	1 (2.0)	
CMTC	2 (0.5)	2 (0.6)	0		0	0		0	0	0	
ATC	3 (0.7)	2 (0.6)	1 (1.1)		0	1 (1.2)		1 (3.4)	0	0	
MTC	4 (0.9)	3 (0.9)	1 (1.1)		0	1 (1.2)		1 (3.4)	0	0	

Abbreviations: ATA, American Thyroid Association; ATC, anaplastic thyroid carcinoma; CMTC, cribriform morular thyroid carcinoma; ETE, extrathyroidal extension; FTC, follicular thyroid carcinomas; IEFVPTC, invasive encapsulated follicular variant papillary thyroid carcinoma; LNM, lymph node metastasis; MTC, medullary thyroid carcinomas; NA, not applicable; NIFTP, noninvasive follicular thyroid neoplasm with papillary-like nuclear features; OCA, oncocytic carcinomas of the thyroid; PTC, papillary thyroid carcinomas; VAF, variant allele fraction.

^a^ The VAF = 0 group was included as an additional group and an overall statistic test comparing all groups was conducted.

^b^ Assessed using χ^2^ or Fisher exact test (2-sided) for categorical variables and 1-way analysis of variance test for independent parametric continuous measures.

^c^ Analysis of 418 nodules for tumor sizes (329 *RAS*-negative and 89 *RAS*-positive nodules) due to lack of information for the missing nodules.

^d^ Analysis of 349 malignant tumors for tumor ETE, LNM, capsular invasion, lymphatic invasion, perineural invasion, and ATA malignancy risk (0, not available; 1, low risk of recurrence; 2, intermediate to high risk of recurrence).

**Table 2.** VAF Assay Findings of Residual Fine-Needle Aspiration Biopsy Specimens From Indeterminate, Malignant, and Benign Thyroid Nodules

Characteristics	Patients, No. (%)	Cytological category						*P* value^a^
		ND	AUS	FN	SFM	Malignant	Benign	
Sample	249 (100)	76 (30.5)	83 (33.3)	26 (10.4)	17 (6.8)	34 (13.7)	13 (5.2)	NA
Sex								
Male	53 (21.3)	20 (26.3)	17 (20.5)	1 (3.8)	1 (5.9)	12 (35.3)	2 (15.4)	.02
Female	196 (78.7)	56 (73.7)	66 (79.5)	25 (96.2)	16 (94.1)	22 (64.7)	11 (84.6)	
Age at biopsy, y								
Mean (SD)	55.5 (15.6)	57.7 (14.2)	56.1 (15.7)	59.0 (13.6)	50.0 (18.6)	50.4 (16.7)	52.4 (17.2)	.10
<55	124 (49.8)	33 (43.4)	42 (50.6)	12 (46.2)	10 (58.8)	19 (55.9)	8 (61.5)	.68
≥55	125 (50.2)	43 (56.6)	41 (49.4)	14 (53.8)	7 (41.2)	15 (44.1)	5 (38.5)	
*RAS* variants								
Absent	213 (85.5)	72 (94.7)	70 (84.3)	24 (92.3)	8 (47.1)	27 (79.4)	12 (92.3)	<.001
Any	36 (14.5)	4 (5.3)	13 (15.7)	2 (7.7)	9 (52.9)	7 (20.6)	1 (7.7)	
* HRAS*	10 (4.0)	0	1 (1.2)	2 (7.7)	4 (23.5)	3 (8.8)	0	<.001
* KRAS*	3 (1.2)	0	2 (2.4)	0	0	1 (2.9)	0	
* NRAS*	23 (9.2)	4 (5.3)	10 (12.0)	0	5 (29.4)	3 (8.8)	1 (7.7)	
*BRAF* V600E variant								
Absent	199 (79.9)	65 (85.5)	66 (79.5)	24 (92.3)	13 (76.5)	18 (52.9)	13 (100)	.001
Present	50 (20.1)	11 (14.5)	17 (20.5)	2 (7.7)	4 (23.5)	16 (47.1)	0	
*TERT* promoter variants								
Absent	224 (90.0)	71 (93.4)	77 (92.8)	19 (73.1)	15 (88.2)	29 (85.3)	13 (100)	.05
Present	25 (10.0)	5 (6.6)	6 (7.2)	7 (26.9)	2 (11.2)	5 (14.7)	0	
*BRAF* and *TERT* variants								
Absent	183 (73.5)	62 (81.6)	62 (74.7)	17 (65.4)	11 (64.7)	18 (52.9)	13 (100)	.005
Present	66 (25.5)	14 (18.4)	21 (25.3)	9 (34.6)	6 (35.3)	16 (47.1)	0	
*BRAF*, *TERT*, and *RAS* variants								
Absent	154 (61.8)	58 (76.3)	50 (60.2)	16 (61.5)	6 (35.3)	12 (35.3)	12 (92.3)	<.001
Present	95 (38.2)	18 (23.7)	33 (39.8)	10 (38.5)	11 (64.7)	22 (64.7)	1 (7.7)	

Abbreviations: AUS, atypia undetermined significance; FN, follicular neoplasm; ND, nondiagnostic; SFM, suspicious for malignancy; and VAF, variant allele fraction.

^a^ χ^2^ or Fisher exact test (2-sided) for categorical variables and 1-way analysis of variance test for independent parametric continuous measures.

### Interpatient Variabilities of *NRAS*, *HRAS*, and *KRAS* Variants in Thyroid Tumors

Molecular VAF assays were developed for the quantitative detection of *RAS* variants at single-nucleotide resolution positive for *NRAS*, *HRAS*, and *KRAS* in tumor tissues but not in the adjacent healthy tissue, which were verified by Sanger sequencing (eFigure 1 in Supplement 1). Of 438 tumors that underwent surgery, 89 (20.3%) were identified with the presence of *RAS* variants, including 51 (11.6%) with *NRAS*, 29 (6.6%) with *HRAS*, and 9 (2.1%) with *KRAS* variants, in mutually exclusive existence from each other (Figure 1 and Table 1). When compared with the 3 *RAS* gene isoforms across all tumor subtypes, the profiles of interpatient variabilities were delineated at the VAF levels ranging from 0.15% to 51.53%, specifically from 0.59% to 51.53% for *NRAS*, from 0.36% to 43.56% for *HRAS*, and from 0.15% to 46.64% for *KRAS* variants, with no significant difference among 3 isoforms (*P* = .16) (Figure 1; eFigure 2 in Supplement 1). Of these variants, 84 (94.4%) exhibited a VAF of greater than 1% and 5 showed a VAF of less than 1%, with 1 *NRAS*, 2 *HRAS*, and 2 *KRAS* variants. *RAS* variants were found in 5 benign neoplasms (6.4%), 5 NIFTPs (41.7%), and 79 malignant neoplasms (22.6%) (*P* < .001). Of 79 malignant neoplasms, 41 (51.9%) were PTCs, from 15.9% of total PTCs; 34 (43.0%) were IEFVPTCs, from 50.7% of total IEFVPTCs; and 4 (5.3%) comprised 1 each of FTCs, OCAs, ATCs, and MTCs, from 17.4% of all these carcinomas (*P* < .001) (Figure 1 and Table 1). Detection of *RAS* variants yielded a sensitivity of 22.6% (95% CI, 18.3%-27.0%), specificity of 88.8% (95% CI, 82.2%-95.3%), positive predictive value (PPV) of 88.8% (95% CI, 82.2%-95.3%), and negative predictive value (NPV) of 22.6% (95% CI, 18.3%-27.0%) in distinguishing malignant neoplasms from benign and NIFTP tumors (eTable 3 in Supplement 1). Notably, the VAF distribution of *RAS* variants was not statistically different among benign, NIFTP, and malignant neoplasms, as well as between PTCs and IEFVPTCs (eFigure 2 in Supplement 1), despite a high incidence of *RAS* variants in both NIFTPs (5 of 12 tumors [41.7%]) and IEFVPTCs (34 of 67 tumors [50.8%]). *RAS* variants, whether at a low or high VAF, were significantly associated with tumors undergoing partial thyroidectomy, with tumors absent for extrathyroidal extension, lymph node metastasis, capsular invasion, lymphatic invasion, or perineural invasion (Table 1). In addition, *RAS* variants alone had a significant association with a low-risk recurrence of thyroid carcinomas (Table 1).

### *RAS*, *BRAF* V600E, and *TERT* Promoter Variants in Thyroid Carcinomas

Of 340 well-differentiated thyroid carcinomas, 77 (22.6%) were detected with *RAS* variants, including 46 (13.5%) with *NRAS*, 25 (7.4%) with *HRAS*, and 6 (1.8%) with *KRAS*. In addition, interpatient variabilities of *BRAF* V600E and *TERT* promoter variants (C228T and C250T) were detected in 173 (50.9%) and 55 (16.2%) carcinomas, respectively, with 45 (13.2%) of them in coexistence. Hence, there were 100 carcinomas (29.4%) with neither *RAS* nor *BRAF* V600E or *TERT* promoter variants (Figure 1; eTable 1 in Supplement 1). *RAS* variants were distributed in 41 PTCs, including 34 classical subtypes (CPTCs), 3 infiltrative follicular subtypes (IFPTCs), and 4 tall, hobnail, or columnar cell subtypes (thcPTCs); 34 IEFVPTCs; and 1 each of FTC and OCA (*P* < .001) (eTable 1 in Supplement 1). Of 41 *RAS* variant PTCs, 6 (14.6%) coharbored *BRAF* V600E alone: 4 in CPTCs and 1 in each of IFPTC and thcPTC; 4 (9.8%) coharbored *TERT* promoter variants alone: 3 in CPTCs and 1 in thcPTC; and 2 (4.9%) coharbored both *BRAF* V600E and *TERT* promoter variants: 1 in each of CPTC and thcPTC. Of 34 *RAS* variant IEFVPTCs, 5 (14.7%) coexisted with *BRAF* V600E alone and 1 (2.9%) coexisted with both *BRAF* V600E and *TERT* promoter variants. For an additional 2 carcinomas with *RAS* variants, 1 in ATC was found coexisting with both *BRAF* V600E and *TERT* promoter variants, and the other in MTC coexisting with *BRAF* V600E alone. Of 57 malignant tumors harboring *RAS* variants alone, 29 (50.9%) were found in PTCs, with 26 CPTCs, 2 IFPTCs, and 1 thcPTC, and 28 (49.1%) were found in IEFVPTCs. No *RAS* variants were detected in the 2 CMTC tumors, but 1 CMTC tumor presented with the coexistence of *BRAF* V600E and *TERT* promoter variants. The inclusion of *RAS* variants into *BRAF* and *TERT* variant assays reached a sensitivity of 70.5% (95% CI, 65.4%-75.2%) and a specificity of 88.8% (95% CI, 79.8%-94.1%), with a PPV of 96.1% (95% CI, 92.7%-98.0%) and an NPV of 43.4% (95% CI, 36.2%-50.9%) in distinguishing malignant neoplasms from benign and NIFTP tumors. This represents a 30.2% increase in sensitivity but a 11.2% decrease in specificity compared with *BRAF* and *TERT* variant assays alone, which had a sensitivity of 54.2% (95% CI, 48.8%-59.4%) and specificity of 100% (95% CI, 94.8%-100%) (eTable 3 in Supplement 1).

### *RAS*, *BRAF* V600E, and *TERT* Promoter Variants in Preoperative Thyroid Nodules

**Figure 3.**
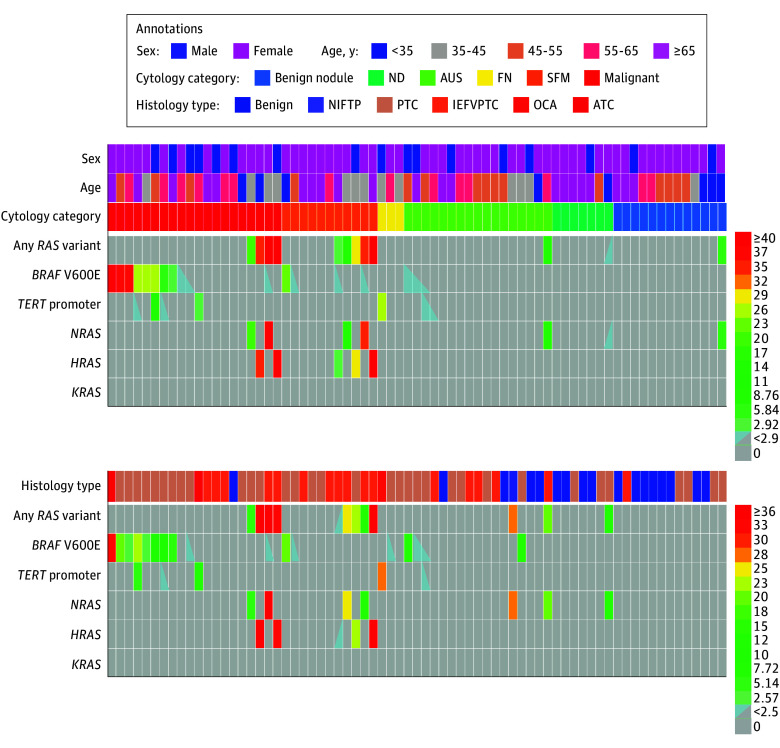
Correlation of Variant Allele Fraction (VAF) Assays of *RAS*, *BRAF* V600E, and *TERT* Promoter Variants Between Residual Fine-Needle Aspiration Specimens and Follow-Up Surgical Thyroid Tumors ATC indicates anaplastic thyroid carcinoma; AUS, atypia of undetermined significance; FN, follicular neoplasm; IEFVPTC, invasive encapsulated follicular variant papillary thyroid carcinoma; ND, nondiagnostic; NIFTP, noninvasive follicular thyroid neoplasm with papillary-like nuclear features; OCA, oncocytic carcinomas of the thyroid; PTC, papillary thyroid carcinomas; and SFM, suspicious for malignancy.

VAF assays of 249 residual FNA specimens identified 36 specimens (14.5%) with *RAS* variants with interpatient variabilities (including 23 FNAs [9.2%] with *NRAS*, 10 FNAs [4.0%] with *HRAS*, and 3 FNAs [1.2%] with *KRAS)*, 50 specimens (20.1%) with *BRAF* V600E, and 25 FNAs (10.0%) with *TERT* promoter variants (Figure 2 and Table 2). Of 36 FNA specimens with *RAS* variants, 28 (77.8%) had *RAS* variants alone in various BSRTC categories (4 ND, 9 AUS, 1 FN, 5 SFM, 5 malignant, and 1 benign); 5 (13.9%) coexisted with *BRAF* V600E: 1 AUS, 2 SFM, and 2 malignant; and 3 (8.3%) coexisted with *TERT* promoter variants: 1 FN and 2 SFM. Interpatient differences in the 5 gene variants (*NRAS, HRAS, KRAS, BRAF*, and* TERT*) were detected in 54 of 126 indeterminate FNAs (42.9%) and 18 of 76 ND FNAs (23.7%). During a median (IQR) follow-up of 88 (50-156) days for patients who underwent resections, VAF assays of 71 residual FNAs achieved a sensitivity of 56.6% (95% CI, 42.4%-69.9%), specificity of 100% (95% CI, 85.9%-100%), PPV of 100% (95% CI, 85.9%-100%), and NPV of 43.9% (95% CI, 28.8%-60.1%) in differentiating malignancy based on their surgical pathological findings (eTable 3 in Supplement 1). Of these FNAs, 12 (16.9%) had *RAS* variants (9 with *RAS* variants alone and 3 coexisting with *BRAF* V600E). Histopathologic outcomes confirmed all 12 (25.4%) nodules were malignant neoplasms, including 5 CPTCs and 7 IEFVPTCs (Figure 3; eTable 2 in Supplement 1). All FNAs with *RAS* variants coexisting with *BRAF* V600E (except for the patient with AUS, who was not available for follow-up) were subsequently found as IEFVPTC. In addition, among 18 nodules (25.4%) identified without *RAS* variants but with *BRAF* V600E or *TERT* promoter variants in the prior FNAs (9 with *BRAF* V600E alone, 3 with *TERT* promoter alone, and 4 in coexistence with both variants), 14 were subsequently found as PTC, with 12 for CPTC and 2 for thcPTC; 2 were found as ATC; and 1 each was found as IEFVPTC and OCA. Among 41 nodules (57.8%) identified with neither *RAS*, *BRAF* V600E, nor *TERT* promoter variants, 17 were benign tumors, 1 was NIFTP, 14 were CPTCs, and 9 were IEFVPTCs. Of note, 1 nodular goiter with an *NRAS* variant in its prior FNA was later confirmed as CPTC. Compared with the 5 gene variants detected in the matched surgical specimens, VAF assays on residual FNA biopsies exhibited a high agreement (κ = 0.799; *P* < .001) (Figure 3) and demonstrated a sensitivity of 87.1% (95% CI, 69.2%-95.8%), specificity of 92.5% (95% CI, 78.5%-98.0%), PPV of 90.0% (95% CI, 72.3%-97.4%), and NPV of 90.2% (95% CI, 75.9%-96.8%).

## Discussion

In this diagnostic study, interpatient variabilities in *RAS* variants were delineated in thyroid tumors with VAFs ranging from 0.15% to 51.53% using sensitive VAF assays. While *RAS* variants alone, regardless of the VAF levels, were associated with thyroid cancer in 88.8% of thyroid nodules harboring such variants, they did not definitively distinguish malignant tumors from NIFTP and benign ones. However, they did facilitate the stratification of low-risk tumors from high-risk ones among malignant neoplasms. Furthermore, interpatient differences in the 5 gene variants were discriminated in 42.9% of indeterminate FNAs, 23.7% ND FNAs, and all FNAs with follow-up surgical pathology-confirmed malignancy.

Currently, molecular assays of *RAS* variants do not effectively risk stratify tumors due to their limited sensitivities and specificities.^27,28^ In our study, the sensitive VAF assays identified substantial interpatient differences in the most common *RAS* gene variants, including 57.3% of *NRAS* variants in predominance, 33.7% of *HRAS* variants, and 9.0% of *KRAS* variants. In a comparable PTC cohort from The Cancer Genome Atlas study, the prevalence of *RAS* variants was 12.9% in PTCs, including 8.5% with *NRAS*, 3.5% with *HRAS*, and 1.0% with *KRAS*, based on NGS assays.^11^ In contrast, our study observed a prevalence of 23.1% for *RAS* variants in PTCs, classified by the 2017 WHO classification,^29^ including 13.5% with *NRAS*, 7.4% with *HRAS*, and 2.2% with *KRAS* (eFigure 3 in Supplement 1), suggesting that VAF assays revealed higher frequencies of *RAS* variants in thyroid neoplasms. Hence, significant discrepancies from different methods of detecting genomic variants may result in false-negative results or missed diagnoses of clinical significance, particularly when methods with lower sensitivities are used.^28,30^ In addition, a high agreement observed in VAF assays between residual FNA biopsies and matched surgical specimens underscores the clinical significance of using residual specimens. At a direct cost of $12.36 per laboratory-developed test reaction coupled with a turnaround time within 8 hours from specimen receipt to result (eTable 4 in Supplement 1), this approach facilitates the timely and rapid delivery of molecular results concurrently with cytological examination on the same source biopsies, holding promise as an effective addition to existing protocols for personalized thyroid cancer care.

High rates of *RAS* variants were identified in lesions exhibiting follicular architecture, such as NIFTP (41.7%) and IEFVPTC (50.7%). It is noteworthy that 70.6% of CPTCs carrying *RAS* variants exhibited a predominantly follicular growth pattern, with most of them presenting encapsulation. Unfortunately, discriminating variant differences did not improve the stratification power of *RAS* variants in distinguishing between malignant neoplasms and NIFTPs, follicular adenomas, or oncocytic adenomas, nor between lesions exhibiting differential follicular architecture, such as NIFTP and IEFVPTC neoplasms. The limited effectiveness of *RAS* variants in stratifying these histological types may be attributed to their close similarity in gene expression profiles.^27,31,32^ Moreover, low VAF events of *RAS* variations, including those at VAF less than 1%, were associated with an equally high risk of cancer as high VAF events. This finding aligns with that of a 2017 study that reported an equivalent malignancy rate in *RAS* variants detected at VAF less than 10% compared with variants detected at VAF greater than 10%.^8^ Further studies are needed to elucidate the biological role and clinical significance of the different extents of *RAS* variations in tumor development.^33,34,35^

The widespread implementation of molecular assays as routine cancer diagnosis remains a challenge. First, interpretation of genomic variations can be complex and may vary due to interpatient differences in such variants.^36,37,38^ *RAS* variants alone, including the low VAF events, do not confirm the malignancy of an unknown tumor; therefore, they should not solely dictate clinical decisions.^39^ However, *RAF* variants do enhance the stratification of low-risk tumors,^12,27^ aiding in informing the extent of operation. Second, *BRAF* V600E and *TERT* promoter variants were detected exclusively in malignant tumors and exhibited a stronger association with aggressive tumor behaviors, aligning with our prior findings and those of other studies.^20,21,40,41^ The inclusion of *RAS* variants into *BRAF* V600E and *TERT* promoter variant assays significantly enhanced the sensitivity for malignancy detection, albeit with a trade-off of reduced specificity. In addition, *RAS* variants coexisting with *BRAF* V600E and/or *TERT* promoter variants tend to be enriched in high-risk cancers, such as thcPTC, FTC, OCA, ATC, and MTC.^42,43,44^ Third, search for novel molecular markers is needed to screen the rest 29.5% of malignant tumors and 64.4% of thyroid nodules that did not have *BRAF* V600E, *TERT* promoter variants, or *RAS* variants. Hence, leveraging NGS with a high-fidelity read capability may help identify additional actionable molecular alterations for detecting malignancy among tumors negative for *RAS, BRAF*, and *TERT* variants.

### Limitations

This study has some limitations. This study was conducted at a single center, where our surgical tumor cohort exhibited a high tumor malignancy rate, potentially contributing to the observed high prevalence of *RAS* variants in malignant tumors. With its ultrasensitivity in absolute quantification, VAF assay stands out as a favorable choice for testing known and definitive biomarkers, be they single variants or a small panel of variants, particularly when dealing with low variant levels. However, VAF assay has a relatively limited capacity of detecting multiple genomic variants in a single reaction. To reinforce the clinical utility of our findings, further larger-scale multicenter validation is necessary, using sensitive VAF assays targeting *RAS* in conjunction with other genomic variants.

## Conclusions

This diagnostic study delineated interpatient variabilities of *RAS* variants in thyroid tumors with various histopathological diagnoses. These findings suggest that discrimination of interpatient differences in *RAS* in combination with *BRAF* V600E and *TERT* promoter variants could facilitate cytology examinations in preoperative precision malignancy diagnosis among patients with thyroid nodules.

## Notes

Supplement 1.
eMethods.
**eTable 1.** Association of Variant Allele Fraction of *RAS* and *BRAF* V600E and *TERT* Promoter Variants With Clinicohistopathologic Features of Well-Differentiated Thyroid Tumors **eTable 2.** Surgical Pathology Follow-up of Variant Allele Fraction Assays of *RAS*, *BRAF* V600E, and *TERT* Promoter Variants on the Residual FNA Biopsy Specimens From Thyroid Nodules **eTable 3.** Performance of Variant Allele Fraction Assays of *RAS* and *BRAF* and *TERT* Variants in Differentiating Malignancy Among Thyroid Surgical Tumors and Preoperative Thyroid Nodules **eTable 4.** Direct Cost of Laboratory Developed Tests for Variant Allele Fraction Assays **eFigure 1.** Detection and Quantification of *HRAS*, *NRAS*, and *KRAS* Variants by dPCR Assays and Verification by Sanger Sequencing **eFigure 2.** Variant Allele Fraction Distributions Across 3 *RAS* Gene Isoforms in Thyroid Tumors and Different Histology Diagnoses **eFigure 3.** Clinicomolecular Characteristics of Interpatient Variabilities of *RAS*, *BRAF* V600E, and *TERT* Promoter Variants at the Variant Allele Fraction Level in Papillary Thyroid Carcinomas (PTC) Classified by The 2017 WHO Classification of Thyroid Neoplasms

Supplement 2.
Data Sharing Statement
