# Supplementary material for: Discriminating Interpatient Variabilities of RAS Gene Variants for Precision Detection of Thyroid Cancer
Source: JAMA Netw Open. 2024 May 17;7(5):e2411919. doi: 10.1001/jamanetworkopen.2024.11919 (PMC11102019; doi:10.1001/jamanetworkopen.2024.11919)
Supplement: Supplement 1. — eMethods. eTable 1. Association of Variant Allele Fraction of RAS and BRAF V600E and TERT Promoter Variants With Clinicohistopathologic Features of Well-Differentiated Thyroid Tumors eTable 2. Surgical Pathology Follow-up of Variant Allele Fraction Assays of RAS, BRAF V600E, and TERT Promoter Variants on the Residual FNA Biopsy Specimens From Thyroid Nodules eTable 3. Performance of Variant Allele Fraction Assays of RAS and BRAF and TERT Variants in Differentiating Malignancy Among Thyroid Surgical Tumors and Preoperative Thyroid Nodules eTable 4. Direct Cost of Laboratory Developed Tests for Variant Allele Fraction Assays eFigure 1. Detection and Quantification of HRAS, NRAS, and KRAS Variants by dPCR Assays and Verification by Sanger Sequencing eFigure 2. Variant Allele Fraction Distributions Across 3 RAS Gene Isoforms in Thyroid Tumors and Different Histology Diagnoses eFigure 3. Clinicomolecular Characteristics of Interpatient Variabilities of RAS, BRAF V600E, and TERT Promoter Variants at the Variant Allele Fraction Level in Papillary Thyroid Carcinomas (PTC) Classified by The 2017 WHO Classification of Thyroid Neoplasms [file jamanetwopen-e2411919-s001.pdf]

Supplemental Online Content

Fu G, Chazen RS, MacMillan C, Witterick IJ. Discriminating interpatient variabilities in *RAS* gene variants for precision detection of thyroid cancer. *JAMA Netw Open*. 2024;7(5):e2411919. doi:10.1001/jamanetworkopen.2024.11919

eMethods.

eTable 1. Association of Variant Allele Fractions of *RAS*, *BRAF* V600E, and *TERT* Promoter Variants With Clinicohistopathologic Features of Well-Differentiated Thyroid Tumors

eTable 2. Surgical Pathology Follow-up of Variant Allele Fraction Assays of *RAS*, *BRAF* V600E, and *TERT* Promoter Variants on the Residual FNA Biopsy Specimens From Thyroid Nodules

eTable 3. Performance of Variant Allele Fraction Assays of *RAS*, *BRAF*, and *TERT* Variants in Differentiating Malignancy Among Thyroid Surgical Tumors and Preoperative Thyroid Nodules

eTable 4. Direct Cost of Laboratory Developed Tests for Variant Allele Fraction Assays

eFigure 1. Detection and Quantification of *HRAS*, *NRAS*, and *KRAS* Variants by dPCR Assays and Verification by Sanger Sequencing

eFigure 2. Variant Allele Fraction Distributions Across 3 *RAS* Gene Isoforms in Thyroid Tumors and Different Histology Diagnoses

eFigure 3. Clinicomolecular Characteristics of Interpatient Variabilities of *RAS*, *BRAF* V600E, and *TERT* Promoter Variants at the Variant Allele Fraction Level in Papillary Thyroid Carcinomas (PTC) Classified by The 2017 WHO Classification of Thyroid Neoplasms

This supplemental material has been provided by the authors to give readers additional information about their work.

© 2024 Fu G et al. *JAMA Network Open.*

eMethods

1.1. Genomic DNA extraction from thyroid tumor tissues and FNA biopsies

Thyroid tumor tissues were subjected to DNA extraction with QIAamp DNA Mini Kit (Qiagen), according to manufacturer's instructions. The remaining material of FNA biopsies preserved in CytoLyte solution (Hologic) were pelleted first, then re-suspended in 200 ul of PBS for DNA isolation based on the protocol of DNA purification from Blood & Body Fluids (Qiagen). The extracted DNA elute was stored at -20 ^{o}C. DNA concentration was measured using NanoDrop 1000 (Thermo Fisher Scientific).

1.2. Droplet dPCR assays of *RAS*, *BRAF* V600E, and *TERT* promoter variants

Molecular assays for 3 *RAS* gene variations, including *NRAS* (Q61R or Q61K), *HRAS* (Q61R or Q61K), and *KRAS* (G12C, G12D, G12V, G12A, or G13D), were developed in-house using locked nucleic acid probe–based NRAS, HRAS, or KRAS Variant Screening Assays (TACT Genomics) on the QX200 AutoDG Droplet dPCR System (Bio-Rad Laboratories) in Central Scientific Laboratory of Lunenfeld-Tanenbaum Research Institute. Briefly, droplet dPCR detection of *RAS* variants was performed in 22 μl reaction mixture containing 11 μl dPCR Supermix for Probes (2×, no dUTP), 1.1 ul of 20x variant screening assay, 3.4 ul of E-solution, 3.0 μl of genomic DNA sample, and 3.5 μl of deionized water. Droplets were generated using a QX200 Droplet Generator the reaction was loaded into the Droplet Generator Cartridge to mix with the Droplet Generation Oil for Probes. The resulting droplets were then transferred to a 96-well PCR plate and PCR amplification was performed in a C1000 Touch™ Thermal Cycler with 96-Deep Well Reaction Module. Amplification conditions were: 1 cycle of 95 °C for 10 min, 40 cycles of 94 °C for 30 s, and 63 °C for *NRAS* and *HRAS* variant assay or 56 °C for *KRAS*

variant assay for 1 min, 1 cycle of 98 °C for 10 min with a ramp rate of 2°C/s, and holding at 4 °C in the end. Similarly, dPCR assays for *BRAF* V600E and *TERT* promoter variants were conducted using BRAF and TERT Variant Screening Assays (TACT Genomics) as previously described.[2,3] By applying a threshold on channel 1 and channel 2 in the 2 dimensional amplitude plot, the droplets were discriminated into separate 6-fluorescein amidite signals in channel 1 (blue dots), separate hexachloro-fluorescein signals in channel 2 (green dots), or fluorescein signals in both channel 1 and 2 (orange dots), or neither fluorescein signals (grey dots), which respectively represented the specific products amplified for the tested gene variant alleles, wild type alleles, both variant and wild type alleles, or no amplification of products. Variant allele fraction (VAF) concentration (C_{VAF}) and wild type allele concentration (C_{WT}) expressed as copies per µl, and VAF abundance (%) were measured using the QX200 droplet reader and analyzed using QuantaSoft Analysis Pro software (Bio-Rad Laboratories). Each test run included positive controls using DNA samples from cell lines or specimens with confirmed *RAS* variants and a negative control using an extraction blank (H2O) to rule out potential contamination. Heatmaps of VAF of genetic variants as well as clinical features and histological subtypes were generated using Heatmap Illustrator (version 1.0). Clinical sensitivity, specificity, positive predictive value, and negative predictive value with 95% confidence intervals were calculated to assess the performance of VAF assays in detecting tumor malignancy in accordance with the Standards for Reporting of Diagnostic Accuracy (STARD) reporting guidelines.[4]

1.3 Sanger sequencing for *RAS* gene variants

PCR and Sanger sequencing were performed to confirm *RAS* variations identified by digital PCR. Fragments of interest were amplified using primers 5′-

© 2024 Fu G et al. *JAMA Network Open.*

TTCATGAAGACCTCACAGTAAA-3′ (forward) and 5′-GGATCCAGACAACTGTTCAAA-3′ (reverse) for *NRAS*, 5′- TTCATGAAGACCTCACAGTAAA-3′ (forward) and 5′-GGATCCAGACAACTGTTCAAA-3′ (reverse) for *HRAS*, and 5′-TTCATGAAGACCTCACAGTAAA-3′ (forward) and 5′-GGATCCAGACAACTGTTCAAA-3′ (reverse) for *KRAS* (Eurofins Genomics). PCR reactions were carried out under thermo-cycling condition of an initial denaturation at 94℃ for 3 min; 40 cycles of denaturation at 95℃ for 15 sec, and annealing and extension at 60℃ for 60 sec; followed by a final extension at 72℃ for 7 minutes using Taq DNA polymerase (Integrated DNA Technologies). PCR products were electrophoresed on 1.5% (wt/vol) agarose gels, purified, and subsequently sequenced using ABI-PRISM 3100 automatic sequencer by Eurofins Genomics (Eurofins Genomics).

Lead contact and method availability

Further information and requests regarding dPCR assays should be directed to and fulfilled by the lead contact, Guodong Fu (David.Fu@sinaihealth.ca; gdfu2002@gmail.com).

References

1. Lloyd RV, Osamura RY, Klèoppel G, Rosai J, WHO, IARC. WHO classification of tumours of endocrine organs, 4th edition. *Lyon, France : International Agency for Research on Cancer (IARC), [2017]*. 2017;tenth volume.
2. Fu G, Chazen RS, Monteiro E, et al. Facilitation of Definitive Cancer Diagnosis With Quantitative Molecular Assays of BRAF V600E and TERT Promoter Variants in Patients With Thyroid Nodules. *JAMA Netw Open.* 2023;6(7):e2323500.
3. Fu G, Chazen RS, MacMillan C, Witterick IJ. Development of a Molecular Assay for Detection and Quantification of the BRAF Variation in Residual Tissue From Thyroid Nodule Fine-Needle Aspiration Biopsy Specimens. *JAMA Netw Open.* 2021;4(10):e2127243.
4. Bossuyt PM, Reitsma JB, Bruns DE, et al. STARD 2015: an updated list of essential items for reporting diagnostic accuracy studies. *BMJ.* 2015;351:h5527.

© 2024 Fu G et al. *JAMA Network Open.*

eTable 1. Association of Variant Allele Fractions (VAF) of *RAS*, *BRAF* V600E, and *TERT* Promoter Variants With Clinicohistopathologic Features of Wide Differentiated Thyroid Tumors

Characteristics	Patients No. (%)	Patients with *RAS* VAF=0	Patients with *RAS* VAF>0	*P value* [a]	*RAS, BRAF, and TERT VAF=0*	*RAS, BRAF, and TERT VAF>0*	*P value* [a]
	340 (100)	263 (77.4)	77 (22.6)		100 (29.4)	240 (70.6)	
**Sex**							
**Male**	92 (27.1)	75 (28.5)	17 (22.1)	*0.308*	29 (29.0)	63 (26.2)	*0.688*
**Female**	248 (72.9)	188 (71.5)	60 (77.9)		71 (71.0)	177 (73.8)	
**Age, y**							
**Mean±SD**	49.1±14.8	49.7±15.0	46.9±14.2	*0.139*	50.2±15.8	48.6±14.4	*0.369*
**< 55**	222 (65.3)	168 (63.9)	54 (70.1)	*0.343*	60 (60.0)	162 (67.5)	*0.211*
**≥ 55**	118 (34.7)	95 (36.1)	23 (29.9)		40 (40.0)	78 (32.5)	
**Thyroidectomy**							
**Partial**	126 (37.1)	87 (33.1)	39 (50.6)	*0.007*	35 (35.0)	91 (37.9)	*0.625*
**Total**	214 (62.9)	176 (66.9)	38 (49.4)		65 (65.0)	149 (62.1)	
**Tumor size, cm**							
**Mean±SD**	2.8±1.7	2.7±1.7	3.0±1.6	*0.180*	3.1±1.8	2.6±1.6	*0.018*
**1 - 2**	146 (42.9)	121 (46.0)	25 (32.5)	*0.088*	31 (31.0)	115 (47.9)	*0.015*
**2 - 4**	135 (39.7)	97 (36.9)	38 (49.4)		47 (47.0)	88 (36.7)	
**> 4**	59 (17.4)	45 (17.1)	14 (18.2)		22 (22.0)	37 (15.4)	
**ETE**							
**None**	304 (89.4)	227 (86.3)	77 (100)	*<.001*	92 (92.0)	212 (88.3)	*0.343*
**Identified**	36 (10.6)	35 (13.7)	0		8 (8.0)	28 (11.7)	
**LNM**							
**None**	235 (69.1)	162 (61.6)	73 (94.8)	*<.001*	76 (76.0)	159 (66.2)	*0.094*
**Identified**	105 (30.9)	101 (38.4)	4 (5.2)		24 (24.0)	81 (33.8)	
**Capsular invasion**							
**None**	245 (72.1)	214 (81.4)	31 (40.3)	*<.001*	67.0 (67.0)	178 (74.2)	*0.187*
**Identified**	95 (27.9)	49 (18.6)	46 (59.7)		33 (33.0)	62 (25.8)	
**Angioinvasion**							
**None**	286 (84.1)	220 (83.7)	66 (85.7)	*0.726*	83 (83.0)	203 (84.6)	*0.746*
**Identified**	54 (15.9)	43 (16.3)	11 (14.3)		17 (17.0)	37 (15.4)	
**Lymphatic invasion**							
**None**	236 (69.4)	164 (62.4)	72 (93.5)	*<.001*	78 (78.0)	158 (65.8)	*0.028*
**Identified**	104 (30.6)	99 (37.6)	5 (6.5)		22 (22.0)	82 (34.2)	
**Perineural invasion**							
**None**	315 (92.6)	238 (90.5)	77 (100)	*0.005*	99 (99.0)	216 (90.0)	*0.005*
**Identified**	25 (7.4)	25 (9.5)	0		1 (1.0)	24 (10.0)	
**AJCC Stage**							
**I**	293 (86.2)	223 (84.8)	70 (90.9)	0.442	90 (90.0)	203 (84.6)	*0.306*
**II**	43 (12.6)	36 (13.7)	7 (9.1)		10 (10.0)	33 (13.8)	
**III**	4 (1.2)	4 (1.5)	0		0	4 (1.7)	
**ATA Malignant Risk**							
**1**	148 (43.5)	94 (35.7)	54 (70.1)	<*.001*	59 (59.0)	89 (37.1)	*0.001*
**2**	155 (45.6)	133 (50.6)	22 (28.6)		33 (33.0)	122 (50.8)	
**3**	37 (10.9)	35 (13.7)	1 (1.3)		8 (8.0)	29 (12.1)	
**WDT tumors**							
**PTC**	258 (75.9)	217 (82.5)	41 (53.5)	*<.001*	68 (68.0)	190 (79.2)	*0.024*
**IEFVPTC**	67 (19.7)	33 (12.5)	34 (44.2)		25 (25.0)	42 (17.5)	
**FTC**	5 (1.5)	4 (1.5)	1 (1.3)		4 (4.0)	1 (0.4)	
**OCA**	10 (2.9)	9 (3.4)	1 (1.3)		3 (3.0)	7 (2.9)	
**PTC**							
**CPTC**	199 (77.1)	167 (66.8)	34 (82.9)	*0.127*	62 (91.2)	137 (72.1)	<*.001*
**IFPTC**	10 (3.9)	7 (3.2)	3 (7.3)		5 (7.4)	5 (2.6)	
**thcPTC**	49 (19.0)	45 (20.7)	4 (9.8)		1 (1.5)	48 (25.3)	

Abbreviations: AJCC: American Joint Committee on Cancer Cancer Staging Manual, 8th Edition; ATA, American Thyroid Association; CPTC, classical subtype of papillary thyroid carcinomas; FTC, follicular thyroid carcinomas; IEFVPTC, invasive encapsulated follicular variant papillary thyroid carcinoma; IFPTC, infiltrative follicular subtype of papillary thyroid carcinomas; OCA,oncocytic carcinomas of the thyroid; PTC, papillary thyroid carcinomas; thcPTC, tall, hobnail, or columnar cell subtypes; and WDT, well differenciated thyroid carcinoma.
[a] Fisher's Exact Test (2-sided) for categorical variables and 1-way ANOVA test for independent parametric continuous measures.

© 2024 Fu G et al. *JAMA Network Open.*

eTable 2. Surgical Pathology Follow-up of Variant Allele Fraction Assays of *RAS*, *BRAF* V600E, and *TERT* Promoter Variants on the Residual FNA Biopsy Specimens From Thyroid Nodules

Characteristics	Patients No. (%)	BSRTC category of Thyroid FNA Biopsy Specimens						*P value* [a]
		ND	AUS	SN	SM	Malignant	Benign	
	71 (100.0)	7 (9.9)	17 (23.9)	3 (4.2)	11 (15.5)	20 (28.2)	13 (18.3)	
**Sex**								
**Male**	17 (23.9)	1 (14.3)	5 (29.4)	1 (33.3)	1 (9.1)	7 (35.0)	2 (15.4)	*0.560*
**Female**	54 (76.1)	6 (85.7)	12 (70.6)	2 (66.7)	10 (90.9)	13 (65.0)	11 (84.6)	
**Age at biopsy, y**								
**Mean±SD**	53.7±14.8	60.9±17.1	52.8±12.2	46.4±13.1	53.6±16.2	54.0±14.6	52.4±17.2	*0.781*
**< 55**	2 (28.6)	8 (61.5)	42 (50.6)	12 (46.2)	10 (58.8)	19 (55.9)	8 (61.5)	*0.714*
**≥ 55**	5 (71.4)	5 (38.5)	41 (49.4)	14 (53.8)	7 (41.2)	15 (44.1)	5 (38.5)	
***RAS* variants**								
**Absent**	59 (83.1)	6 (85.7)	16 (94.1)	3 (100)	6 (54.5)	16 (80.0)	12 (92.3)	*0.131*
**Any**	12 (16.9)	1 (14.3)	1 (7.7)	0	5 (45.5)	4 (20.0)	1 (7.7)	
HRAS	5 (7.0)	0	0	0	3 (27.3)	2 (10.0)	0	
KRAS	0	0	0	0	0	0	0	*0.319*
NRAS	7 (9.9)	1 (14.3)	1 (7.7)	0	2 (18.2)	2 (10.0)	1 (7.7)	
***BRAF* V600E**								
**Absent**	53 (74.6)	7 (100)	14 (82.4)	3 (100)	7 (63.6)	9 (45.0)	13 (100)	*0.002*
**Present**	18 (25.4)	0	3 (17.6)	0	4 (36.4)	11 (55.0)	0	
***TERT* promoter variants**								
**Absent**	64 (90.1)	7 (100)	15 (88.2)	2 (66.7)	11 (100)	16 (80.0)	13 (100)	*0.173*
**Present**	7 (9.9)	0	2 (11.8)	1 (33.3)	0	4 (20.0)	0	
***BRAF* and *TERT* variants**								
**Absent**	50 (70.4)	7 (100)	13 (76.5)	2 (66.7)	7 (63.6)	8 (40.0)	13 (100)	*0.001*
**Present**	21 (29.6)	0	4 (23.5)	1 (33.3)	4 (36.4)	12 (60.0)	0	
***BRAF*, *TERT*, and *RAS* variants**								
**Absent**	41 (57.7)	6 (85.7)	12 (70.6)	2 (66.7)	4 (36.4)	5 (25.0)	12 (92.3)	*<.001*
**Present**	30 (42.3)	1 (14.3)	5 (29.4)	1 (33.3)	7 (63.6)	15 (75.0)	1 (7.7)	
**Malignant**								
**Benign**	18 (25.4)	4 (57.1)	5 (29.4)	0	0	1 (5.0)	8 (61.5)	*<.001*
**Malignant**	53 (74.6)	3 (42.9)	12 (70.6)	3 (100)	11 (100)	19 (95.0)	5 (38.5)	

Abbreviations: AUS, atypia undetermined significance; FN, follicular neoplasm; ND, non-diagnostic; and SFM, suspicious for malignancy.

[a] Fisher's Exact Test (2-sided) for categorical variables and 1-way ANOVA test for independent parametric continuous measures.

© 2024 Fu G et al. *JAMA Network Open.*

eTable 3. Performance of Variant Allele Fraction (VAF) Assays of *RAS*, *BRAF*, and *TERT* Variants in Differentiating Malignancy Among Thyroid Surgical Tumors and Preoperative Thyroid Nodules

**VAF Assays**	***RAS* Variants**	***BRAF* and *TERT* Variants**	***RAS, BRAF, and TERT* variants**	
**Samples**	Surgical tumors	Surgical tumors	Surgical tumors	Thyroid nodules
**True positive** [a]	79 (18.0)	189 (43.2)	246 (56.2)	30 (42.3)
**False positive** [a]	10 (2.3)	0 (0)	10 (2.3)	0 (0)
**True negative** [a]	79 (18.0)	89 (20.3)	79 (18.0)	18 (25.4)
**False negative** [a]	270 (61.6)	160 (36.5)	103 (23.5)	23 (32.4)
**Sensitivity** [b]	22.6 (18.4, 27.5)	54.2 (48.8, 59.4)	70.5 (65.4, 75.2)	56.6 (42.4, 69.9)
**Specificity** [b]	88.8 (79.9, 94.2)	100 (94.8, 100)	88.8 (79.9, 94.2)	100 (78.1, 100)
**PPV** [b]	88.8 (79.9, 94.2)	100 (97.5, 100)	96.1 (92.7, 98.0)	100 (85.9, 100)
**NPV** [b]	22.6 (18.4, 27.5)	35.7 (29.9, 42.1)	43.4 (36.2, 50.9)	43.9 (28.8, 60.1)

Abbreviations: NPV, negative predictive value; and PPV, positive predictive value.
[a] Data are presented as numbers (percentages) of cases.
[b] Data are presented as estimated percentages (95% confidence interval).

© 2024 Fu G et al. *JAMA Network Open.*

eTable 4. Direct Cost of Laboratory Developed Tests for Variant Allele Fraction (VAF) Assays

Description	Cost/reaction (US$)	Turnaround (hours)
**DNA extraction**<br>(Genomic DNA isolation Kit and concentration measurement)	$3.24	≤ 2.5
**dPCR** & **data acquisition**<br>(consumables, reagents, and variant assays)	$9.12	≤ 4.5
**Data analysis and report**<br>(Variant, wild type and VAF calculation, and assay report)		≤ 1.0
**In total**	$12.36	≤ 8.0

Note: Calculations of direct cost of laboratory developed dPCR assays were based on the cost of key consumables for DNA isolation kits, variant screening assays, and dPCR reagents, and the turnaround time calculations were based on triplicate dPCR assays of 15 samples. However, it's important to note that indirect costs that we did not include, such as human capital (personnel costs), assay development and validation, and facility-related expenses, are significant components of the total cost of molecular tests.

© 2024 Fu G et al. *JAMA Network Open.*

eFigure 1

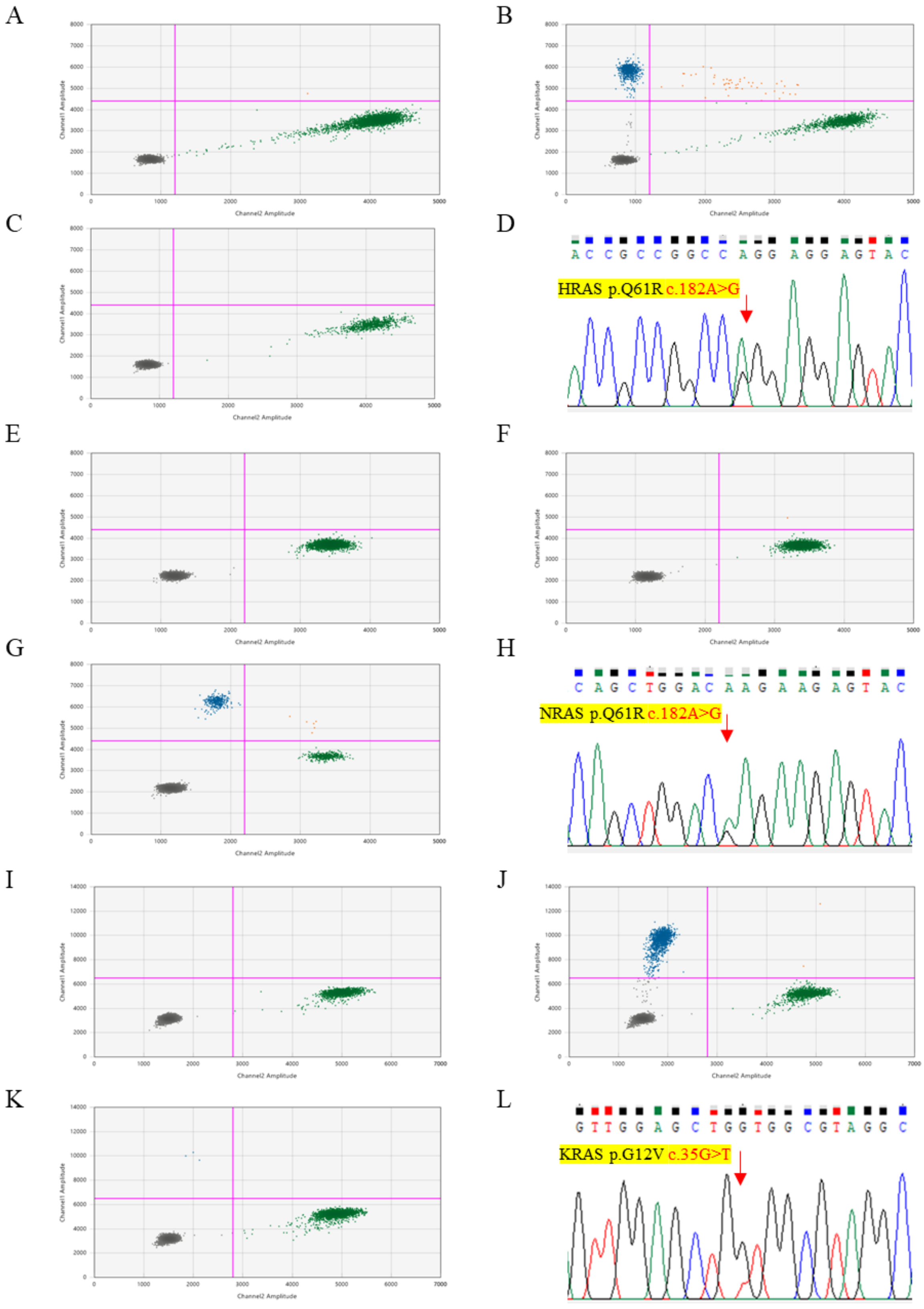

© 2024 Fu G et al. *JAMA Network Open.*

eFigure 1. Detection and Quantification of *HRAS*, *NRAS*, and *KRAS* Variants by dPCR Assays Followed by Verification through Sanger Sequencing. A, *HRAS* wild type detected in FTC-133 cells. B. *HRAS* p.Q61R c.182A>G in tumor tissue of specimen #23505 detected and quantified as a variant allele fraction (VAF) of 34.77%. C, *HRAS* wild type detected in tumor tissue of specimen #23680. D. Sequencing electropherograms of *HRAS* p.Q61R c.182A>G in tumor tissue of specimen #23505. E, *NRAS* wild type detected in FTC-133 cells. F. *NRAS* wild type detected in tumor tissue of specimen #23505. G, *NRAS* p.Q61R c.182A>G in specimen #23680 detected and quantified as a VAF of 43.19%. H. Sequencing electropherograms of *NRAS* p.Q61R c.182A>G in tumor tissue of specimen #23680. I, *KRAS* wild type detected in FTC-133 cells. J. *KRAS* p.G12V c.35G>T in tumor tissue of specimen #23172 detected and quantified as a VAF of 39.40%. K, *KRAS* wild type detected in the tumor adjacent health tissue of specimen #23172. L. Sequencing electropherograms of *KRAS* p.G12V c.35G>T in specimen #23172. 2 dimensional plots of *HRAS*, *NRAS*, and *KRAS* dPCR variant assays were established with threshold (red line) at both Channel-1 and Channel-2 to classify positive and negative droplets. Positive droplets in Channel-1 (blue dots) indicated the specific binding of the locked nucleic acid 6-fluorescein amidite variant probes to the specific *RAS* variation and positive droplets in Channel-2 (green dots) indicated the specific binding of locked nucleic acid hexachloro-fluorescein wild-type probes to the wild-type *RAS*, while negative droplets were displayed in gray. Representative sequencing electropherograms of Sanger sequencing was performed to verify the target specific *RAS* variant or its wild-type identified by dPCR.

© 2024 Fu G et al. *JAMA Network Open.*

eFigure 2

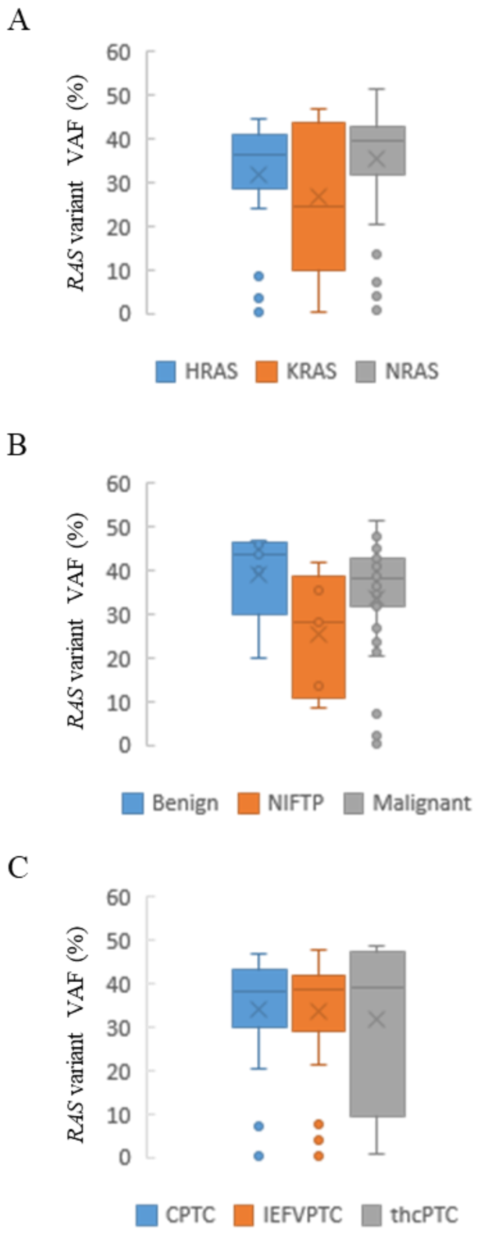

eFigure 2. Variant Allele Fraction (VAF) Distributions Across 3 *RAS* Gene Isoforms in Thyroid Tumors and Different Histological Diagnoses. (**A**) Box plots of VAF (%) distribution in three differentially expressed *RAS* gene isoforms, *NRAS*, *HRAS*, and *KRAS* variants (P = 0.162). (**B&C**) Box plots of VAF (%) distribution of *RAS* variants (*NRAS*, *HRAS*, and *KRAS* in combination) across benign, NIFTP, and malignant tumors (P = 0.278) (**B**) and cPTC, IEFVPTC, and thcPTC (P = 0.951) (**C**). One-way analysis of variance test showed no significant difference among groups compared based on $P < 0.05$. CPTC indicates classical subtype of papillary thyroid carcinomas; IEFVPTC, invasive encapsulated follicular variant papillary thyroid carcinoma; NIFTP, noninvasive follicular thyroid neoplasm with papillary-like nuclear features; and thcPTC, tall, hobnail, or columnar cell subtypes.

© 2024 Fu G et al. *JAMA Network Open.*

eFigure 3

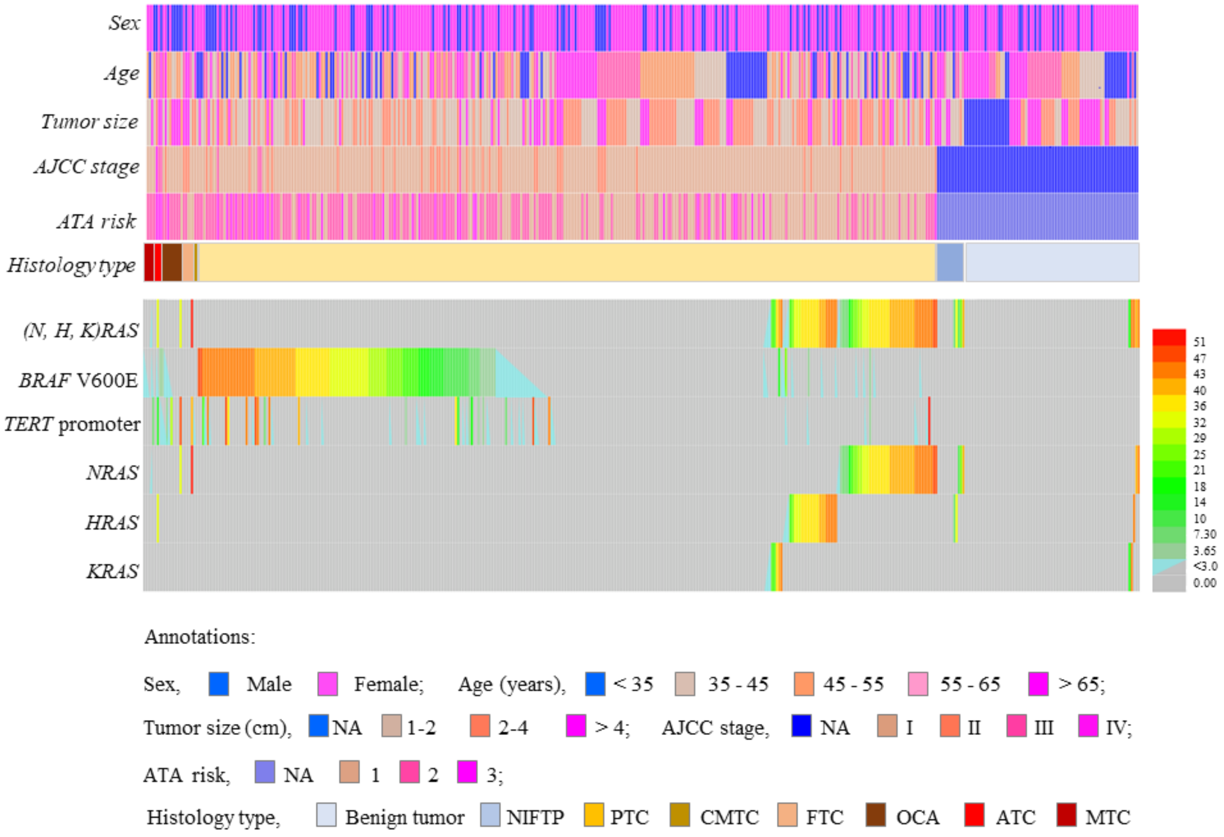

eFigure 3. Clinicomolecular Characteristics of Interpatient Variabilities of *RAS*, *BRAF* V600E, and *TERT* Promoter Variants at the Variant Allele Fraction (VAF) Level in Papillary Thyroid carcinomas (PTC) Classified by The 2017 WHO Classification of Thyroid Neoplasms. The 325 PTCs were classified according to 2017 WHO classification by which IEFVPTC was classified into the group of follicular variant of PTC (FVPTC), comprising 199 (61.2%) classical PTC (CPTC), 77 (23.7%) FVPTC, and 49 (15.1%) tall, hobnail, or columnar cell subtypes (thcPTC). AJCC indicates American Joint Committee on Cancer Cancer Staging Manual, 8th Edition; ATA, American Thyroid Association; ATC, anaplastic thyroid carcinoma; CMTC, cribriform morular thyroid carcinoma; FTC, follicular thyroid carcinomas; IEFVPTC, invasive encapsulated follicular variant papillary thyroid carcinoma; MTC, medullary thyroid carcinomas; NIFTP, noninvasive follicular thyroid neoplasm with papillary-like nuclear features; and OCA, oncocytic carcinomas of the thyroid.

© 2024 Fu G et al. *JAMA Network Open.*
