# Supplementary material for: Discriminating Interpatient Variabilities of RAS Gene Variants for Precision Detection of Thyroid Cancer
Source: JAMA Netw Open. 2024 May 17;7(5):e2411919. doi: 10.1001/jamanetworkopen.2024.11919 (PMC11102019; doi:10.1001/jamanetworkopen.2024.11919)
Supplement: Supplement 2. — Data Sharing Statement [file jamanetwopen-e2411919-s002.pdf]

Data Sharing Statement

Fu. Discriminating Interpatient Variabilities of RAS Gene Variants for Precision Detection of Thyroid Cancer. *JAMA Netw Open*. Published May 17, 2024.
doi:10.1001/jamanetworkopen.2024.11919

Data
Data available: No

Additional Information
Explanation for why data not available: Based upon our REB approval, we may share de-identified aggregated data only, and therefore cannot share individual patient data.
